# A Mathematical Model for Wind Velocity Field Reconstruction and Visualization Taking into Account the Topography Influence

**DOI:** 10.3390/jimaging10110285

**Published:** 2024-11-07

**Authors:** Guzel Khayretdinova, Christian Gout

**Affiliations:** 1 National Institute for Applied Sciences (INSA Rouen Normandie), Laboratoire de Mathématiques de l’INSA, LMI-UR 3226, 76000 Rouen, France; 2 CNRS, Normandie Mathématiques, FR CNRS 3335, 76000 Rouen, France; 3 Department of Mathematics, University of Hawai’i at Manoa, 2565 McCarthy Mall, Keller Hall, Honolulu, HI 96822, USA

**Keywords:** vector flow visualization, current/wind velocity field approximation, wind velocity field modelling

## Abstract

In this paper, we propose a global modelling for vector field approximation from a given finite set of vectors (corresponding to the wind velocity field or marine currents). In the modelling, we propose using the minimization on a Hilbert space of an energy functional that includes a fidelity criterion to the data and a smoothing term. We discretize the continuous problem using a finite elements method. We then propose taking into account the topographic effects on the wind velocity field, and visualization using a free library is also proposed, which constitutes an added value compared to other vector field approximation models.

## 1. Introduction

Vector field approximation has many applications, such as to predict wind turbine production; in oceanography, to study marine currents; and more generally in a computer sciences framework. As introduced in [1], in order to approximate a vector field, several approaches have been developed: a finite element method interpolation (see [2]), PDE-based methods, kriging methods, a Lagrange interpolation method and spline and Rational Basis Function (RBF) approximations (see [3,4,5,6,7,8]). These approaches have drawbacks, particularly when a small number of data are available and the approximation’s result is qualitatively insufficient. In this work, we are precisely in a case where the number of data from anemometers is considered low in comparison to the large study area. It is, therefore, necessary to propose a mathematical model allowing for this type of data to be processed via a robust energy functional minimization. The major methodological contribution of this work consists in the modelling using D^m^ splines, as well as the contribution of adding the topography effect into the numerical results; most of the methods proposed in the literature do not integrate this important aspect, but because there are little available data, it is important to include as much information in the model as possible to generate a realistic wind field, and taking the topography into account is quite simple and brings significant added value. Other methods (like [3,4,5]) only focus on the modelling and the mathematical aspects of the approximation without adding more information than the input dataset. To our knowledge, our approach is the first to offer such a global framework.

**Figure 1:**
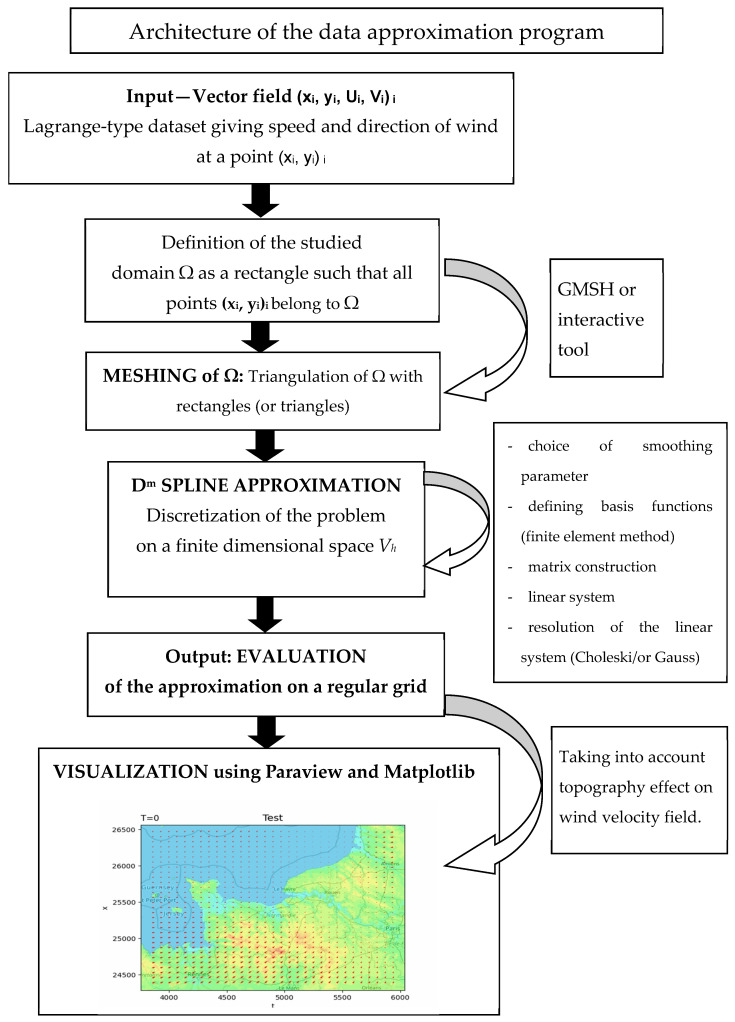
Global view of the approximation framework.

In this paper, we first give the considered vector field approximation model, using a D^m^ spline operator, rigorously introduced in [1,7,9,10,11]. The dataset consists of a finite set of vectors (xi, yi, wi)_i_, where (xi, yi) locates the point in 2D and wi = (Ui, Vi) ∈ *R*^2^ gives the direction and speed of the wind at location (xi, yi). A minimization problem is introduced, leading to a variational problem whose solution is the searched for wind vector field. We give the discretization using a classic finite element method. We then give details on how to integrate into the model the effect of the topography on the obtained wind velocity field; this last part greatly improves upon previous models (given in [1]), where topographic effects are not taken into account. We show the effects of this topographic influence on the synthetic dataset given by an explicit function. We then give numerical examples for a real dataset, including a specific tool for visualization. A global review of this complete approximation framework is given in Figure 1.

## 2. Mathematical Modelling

The mathematical modelling of our approximation problem is constructed using a D^m^ spline operator as follows: For all $v\in H^{m+1}\left(\Omega \right)$, we introduce the energy functional consisting of two terms. The first one is the data fidelity criterion, while the second one is the smoothing parameter:

(1) $$J_{\varepsilon }\left(v\right)={\langle \rho \left(v-w\right)\rangle }^{2}+{\varepsilon {\left|v\right|}^{2}}_{m+1,\Omega ,R}$$

where Ω is an open subset corresponding to the studied zone. *w = (w_1_, w_2_, …, w_N_)* $\in {\left(R^{2}\right)}^{N}$ is the vector field dataset, ρ is a linear operator linked to the dataset, ${{\left|•\right|}^{2}}_{m+1,\Omega ,IR}$ is the usual semi-norm of the usual Sobolev space H*^m^*^+1^(Ω) as defined in [1], $\langle •\rangle$ is the Euclidean norm in R^2^ and *ε* is a smoothing parameter generally equal to 10^−6^ in many applications (see [1,4,7,8] for more details). We recall that H*^m^*^+1^(Ω) is a space of functions belonging to L^2^(Ω) and their (*m* + 1) derivatives. We also introduce an ordered set of *N* points *(x_i_)_i_* of Ω, where we suppose as known the value of the wind velocity field. The linear operator ρ is given by $\rho (v)=(v(x_{1}),v(x_{2}),\ldots v(x_{N}))\in {\left(R^{2}\right)}^{N}.$ We use the D^m^ spline (see Gout et al. [1] and Arcangéli et al. [7] for a complete study of this approximation operator) approximation framework to solve this problem. We call $\sigma_{\varepsilon }^{d}$ the smoothing spline on Ω relative to ρ, which is the unique solution of the minimization problem:

(2) $$\left\{\begin{array}{c} \;\mathrm{find}\;\sigma_{\varepsilon }\in H^{m+1}(\Omega ),\;\mathrm{such}\;\mathrm{that}\;\mathrm{for}\;\mathrm{any}\;v\;\in {\;H}^{m+1}(\Omega ):\\ J_{\varepsilon }\left(\sigma_{\varepsilon }\right)\leq J_{\varepsilon }\left(v\right). \end{array}\right.$$

We can use the Lax–Milgram theorem to establish the uniqueness of this minimization problem, since the solution $\sigma_{\varepsilon }^{d}$ of this minimization problem is the solution of the following variational problem

(3) $$\left\{\begin{array}{c} \mathrm{find}\;\sigma_{\varepsilon }\in H^{m+1}(\Omega ),\;\mathrm{such}\;\mathrm{that}\;\mathrm{for}\;\mathrm{any}\;v\;\in {\;H}^{m+1}(\Omega ):\\ \langle \rho \sigma_{\varepsilon }^{d},\rho v\rangle +\varepsilon {\left(\sigma_{\varepsilon }^{d},v\right)}_{m+1,\Omega ,R}=\langle w,\rho v\rangle . \end{array}\right.$$

where ${\left(•,•\right)}_{m+1,\Omega ,R}$ denotes the semi-norm of $H^{m+1}\left(\Omega \right)$. To apply the Lax–Milgram theorem, we recall that all the hypotheses of this theorem are satisfied, since $H^{m+1}(\Omega )$ is a Hilbert space, and we also have as follows:

- $a(u,v)=\langle \rho u,\rho v\rangle +\varepsilon {\left(u,v\right)}_{m+1,\Omega ,R}$ is a bilinear form, being the sum of scalar products.
- *a(u*,*v)* is continuous on ${\left(H^{m+1}\left(\Omega \right)\right)}^{2}$ because $\left|a(u,v\right|)\leq \max(1,\varepsilon ){\left\|u\right\|}_{m+1,\Omega }{\left\|v\right\|}_{m+1,\Omega }$, using the Cauchy–Schwarz inequality and the norm equivalence between ${\left(\langle \rho v,\rho v\rangle +{\left(v,v\right)}_{m+1,\Omega ,R}\right)}^{1/2}$ and ${\left\|v\right\|}_{m+1,\Omega }$.
- *a(v*,*v)* is elliptic on $H^{m+1}\left(\Omega \right)$ since $a(v,v)\geq \min(1,\varepsilon ){\left\|v\right\|}^{2}m+1,\Omega .$
- $\langle w,\rho v\rangle$ is a continuous linear form.

We now propose a discretization of the variational problem using a finite element discretization (see [1,9,12] for more details on such a discretization). We recall that the main idea of the finite element method is to replace the (Hilbert) space $H^{m+1}(\Omega )$ used to define the variational Equation (3) by a finite dimensional subspace V_h_. Of course, we have $V_{h}\subset H^{m+1}(\Omega )$. The functions belonging to V*_h_*. are piecewise polynomials, and the bases of the functions for the space V*_h_* are constructed such that they have small support. For any real *h > 0*, let *T_h_* be a triangulation of Ω by *n*-simplices or *n*-rectangles *K* with diameter *h_K_* ≤ *h.* We classically approximate the space H*^m^*^+1^(Ω) by the space V*_h_*_,_, a finite dimensional space included in H*^m^*^+1^(Ω) and admitting a polynomial basis of polynomial functions ${\left(\Phi_{j}\right)}_{j}$. We write the solution of $\sigma_{\varepsilon ,h}^{d}$ on the basis of V*_h_* as

(4) $$\sigma_{\varepsilon ,h}^{d}=\sum_{j=1}^{\dim V_{h}}\beta_{j}\Phi_{j}\;:\;\;\beta_{j}\in R.$$

Note that the polynomials ${\left(\Phi_{j}\right)}_{j}$ are given, since they are computed following the chosen generic finite element. The generic finite element we choose here is the Bogner–Fox–Schmit (BFS) rectangle of class *C*^1^, where a function of *V^h^*. is completely determined by its four values (value, values of the two first derivatives and value of the twist derivative) at a nodal point (see Appendix A). The choice of the BFS finite element is due to their capability to easily tessellate rectangular domains and to guarantee a final approximation of class *C*^1^.

From (4), we have to find ${\left(\beta_{j}\right)}_{j}$ in order to find the solution $\sigma_{\varepsilon ,h}^{d}$ of our approximation problem. We can now give the discretization of problem (3) using (4):

(5) $$\begin{array}{l} Find\;{\left(\beta_{j}\right)}_{j}\in \;R^{\dim V_{h}}\;such\;\;that\;\forall k=1,\ldots ,\dim V_{h},\\ \sum_{i=1}^{N}\langle \sum_{j=1}^{\dim V_{h}}\beta_{j}.\Phi_{j},v_{h}(x_{i})\rangle +\varepsilon {\left(\sum_{j=1}^{\dim V_{h}}\beta_{j}.\Phi_{j},v_{h}\right)}_{m+1,\Omega ,R^{n}}=\sum_{i=1}^{N}\langle w_{i},v_{h}(x_{i})\rangle . \end{array}$$

Equation (5) leads to the following linear system, taking as the test function $v_{h}$ all the basis functions $\Phi_{k}$: *k =* 1,*…*, *dim V_h_*

(6) $$\sum_{i=1}^{N}\sum_{j=1}^{\dim V_{h}}\beta_{j}.\Phi_{j},\Phi_{k}(x_{i})+\varepsilon \sum_{j=1}^{\dim V_{h}}\beta_{j}.{\left(\Phi_{j},\Phi_{k}\right)}_{m+1,\Omega ,R^{n}}=\sum_{i=1}^{N}\langle w_{i},\Phi_{k}(x_{i})\rangle .$$

We finally have to solve the following linear system to find the unknown real values ${\left(\beta_{j}\right)}_{j}$:

(7) $$\left({\left(A\right)}^{T}A+\varepsilon R\right)\beta ={\left(A\right)}^{T}w,\;\mathrm{with}\;A={\left[\Phi_{j}(x_{i})\right]}_{1\leq i\leq N,1\leq j\leq \dim V_{h}}\;\mathrm{and}\;R={\left[{\left(\Phi_{j},\Phi_{j}\right)}_{m+1,\Omega ,R}\right]}_{1\leq i,j\leq \dim V_{h}}.$$

For the numerical simulation, we take m = 1, and we use the Bogner–Fox–Schmit finite element with the basis function as a polynomial of degree 3 (see [7] for more details). The modelling we have introduced in this section permits the approximation of a wind velocity field on all Ω from a finite set of data given, for instance, by several anemometers (as illustrated in the numerical section of this work—Figure 10 and Figure 11).

## 3. Taking into Account the Topography

**Figure 2:**
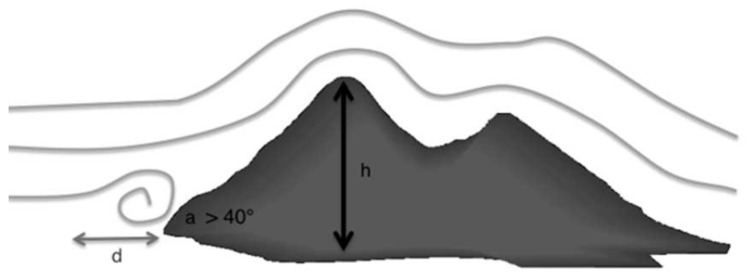
Horizontally, we consider that air streams begin to rise upstream of an obstacle at a distance such that d = h × cot(a/2), with h being the height of the obstacle and *a* the angle of the slope.

In Section 2, we proposed a framework to approximate a wind velocity field from a finite set of measures. It is, of course, well known that the topography plays an important role in wind field velocity variations. Obstacles modify air flows due to pressure forces (see Figure 2). The wind slows down upstream of an obstacle, and accelerates downstream of it.

Since the approach introduced in Section 2 does not take into account physical considerations, we propose here a way to approximate the topography’s influence by post processing the approximated wind field obtained from the model given in Section 2. More precisely, let us consider a wind vector field on N points as

**Figure 3:**
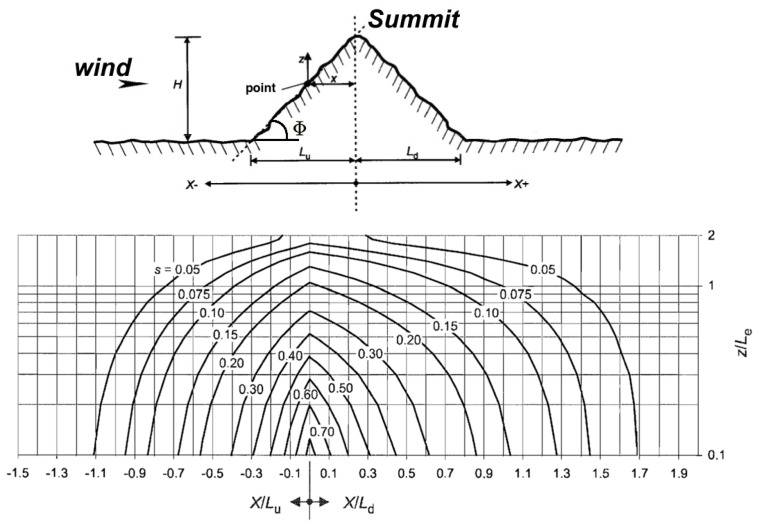
**Top**: considered parameters for hills and ridges (source: [13]). **Bottom**: corresponding values of parameter *s*.

W’ = (w’_1_,…, w’_N_),

where each wind vector w’_i_ belongs to R x R, with given coordinates (x_i_, y_i_) for each w’_i_, according to the topographic configuration around it as follows: W’_i_ = c_i_(θ) w_i,_ where W’_i_ is the adjusted wind vector and c_i_(θ) is the coefficient computed from the topographic configuration at point (x_i_, y_i_), depending on the wind direction θ. This approximation holds for local topographic effects. It cannot take into account large-scale effects, such as Venturi effects in valleys or straits. To compute the topographic coefficient c_i_ for a given wind direction θ at point (x_i_, y_i_), we used the formulas given in parts 1–4 of [13], depending on the slope Φ

(8) $$\left\{\begin{array}{ll} 1, & \Phi <0.05\\ 1+2s\Phi , & 0.05<\Phi <0.3\\ 1+0.6s, & \Phi >0.3 \end{array}\right.$$

where s is the characteristic coefficient of the obstacle, depending on its features (see Table 1 and Figure 3).

The effective length L_e_ is computed as follows (type of slope *Φ = H/L_u_*):

(9) $$L_{e}=\left\{\begin{array}{c} L_{u},\;0.05<\Phi <0.3\\ \frac{H}{0.3},\;\;\;\;\;\;\;\;\;\;\;\;\Phi >0.3. \end{array}\right.\;$$

We also have to compute the value of the orographic location factor *s* used in (8). As shown in [13], the value of s is related to the ratio *H/L_e_*. More precisely, for an upwind section, for ranges $-1.5\leq \frac{x}{L_{u}}\leq 0\;\;\mathrm{and}\;\;0\leq \frac{z}{L_{e}}\leq 2\;$ we take $s=A\exp\left(B\frac{x}{L_{u}}\right),\;$ where

$$A=0.1552{\left(\frac{z}{L_{e}}\right)}^{4}-0.8575\;{\left(\frac{z}{L_{e}}\right)}^{3}+1.8133{\left(\frac{z}{L_{e}}\right)}^{2}-1.9115\left(\frac{z}{L_{e}}\right)+1.0124,$$

$\mathrm{and}\;B=0.3542{\left(\frac{z}{L_{e}}\right)}^{2}-1.0577\left(\frac{z}{L_{e}}\right)+2.6456.$ Note that when $\frac{x}{L_{u}}\leq -1.5\;\;\mathrm{or}\;\;2\leq \frac{z}{L_{e}}\;$ we take *s =* 0.

For a downwind section, as shown in [13], we take $s=A{\left(\log\frac{x}{L_{e}}\right)}^{2}+B\log\frac{x}{L_{e}}+C,\;$ with

$$A=-1.342{\left(\log\frac{z}{L_{e}}\right)}^{3}-0.822\;{\left(\log\frac{z}{L_{e}}\right)}^{2}+0.4609\log\frac{z}{L_{e}}-0.0791,$$

$$B=-1.0196{\left(\log\frac{z}{L_{e}}\right)}^{3}-0.891{\left(\log\frac{z}{L_{e}}\right)}^{2}+0.5343\log\frac{z}{L_{e}}-0.1156,$$

$$\mathrm{and}\;\;C=0.803{\left(\log\frac{z}{L_{e}}\right)}^{3}+0.4236{\left(\log\frac{z}{L_{e}}\right)}^{2}-0.5738\log\frac{z}{L_{e}}+0.1606.$$

**Figure 4:**
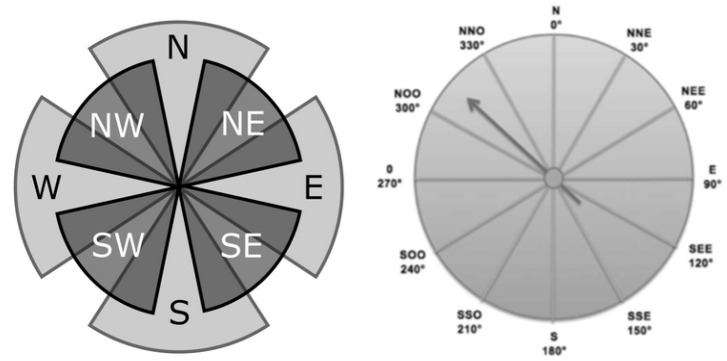
Usual examples of compass wind and wind rose.

To compute the topographic coefficients of a domain D = [0, 1]^2^ × R^2^, we consider a regular grid D_h_ of D of step h. Then, for each point (x_i_, y_i_) = (ih, jh) in D_h_, we compute a coefficient for each wind direction θ. We split up the compass wind into eight directions θ_j_ from 0 to 360°, by steps of 45 degrees (see Figure 4).

Once the collection of topographic coefficients is computed using (8), we use it to adjust the approximated wind field, selecting coefficients according to the direction of each wind vector and using them on the obtained approximating wind velocity field.

## 4. Numerical Examples

In this section, we give several numerical examples, including the computation of the topographic coefficients (using a given function *f*) and the approximation of a vector field from a finite set of vectors giving the direction and speed of the wind.

### 4.1. Computation of the Topographic Coefficients

In order to illustrate the proposed methodology on synthetic data given by an explicit given function *f*, we simulate an obstacle (hills) in domain *D* using the basic 2D function *f* defined as follows:

(10) $$\begin{array}{c} f(x,y)=\frac{3}{4}\exp\left(-\frac{1}{4}{\left(-9x-2\right)}^{2}-\frac{1}{4}{\left(9y-2\right)}^{2}\right)\\ +\frac{3}{4}\exp\left(-\frac{1}{49}{\left(-9x+1\right)}^{2}-\frac{1}{4}{\left(9y+1\right)}^{2}\right)\\ +\frac{1}{2}\exp\left(-\frac{1}{4}{\left(-9x-7\right)}^{2}-\frac{1}{4}{\left(9y-3\right)}^{2}\right)\\ -\frac{1}{5}\exp\left(-\frac{1}{4}{\left(-9x-4\right)}^{2}-\frac{1}{4}{\left(9y-7\right)}^{2}\right) \end{array}$$

**Figure 5:**
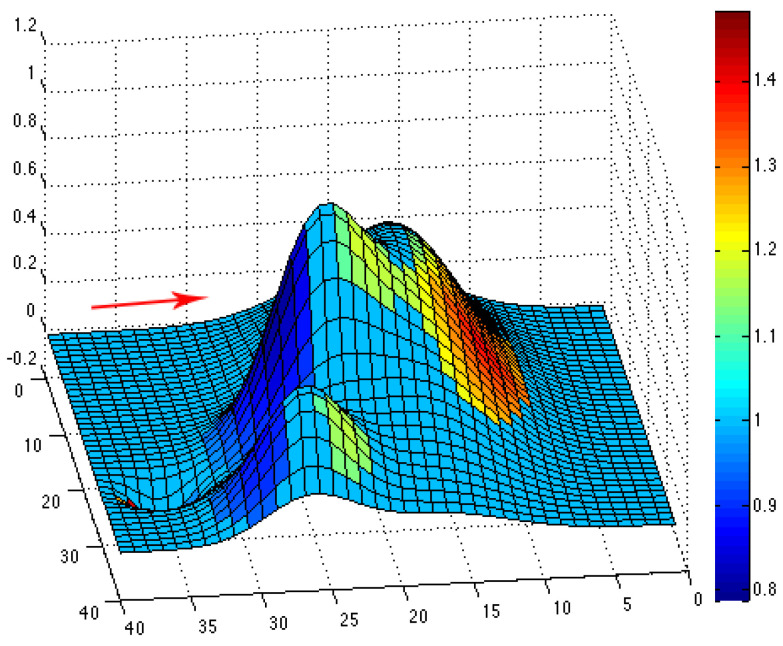
Example of an obstacle given by the function *f* in (10); the arrow gives the considered wind direction (eastern wind). We also give the colormap of the topographic coefficients associated with the east wind direction.

We define the discretized domain as D_h_, with *h =* 1/*n*. The obstacle is obtained by computing *f* for every couple point (x_i_, y_i_), where i, j = 0, …, *n*. For *n* = 40, we obtain the following obstacle (see Figure 5), and we give on this image the computed topographic coefficients obtained using (10) (considering an arbitrary wind direction indicated by the red arrow).

**Figure 6:**
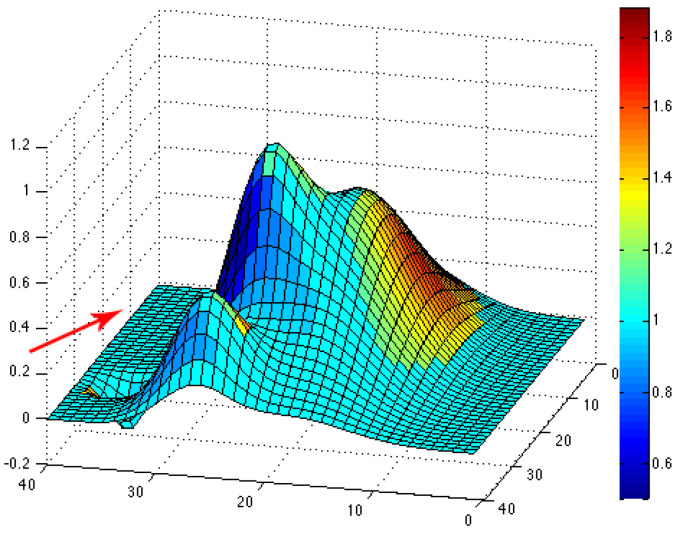
Color map of topographic coefficients associated with a northeast wind direction. The arrow gives the considered wind direction.

**Figure 7:**
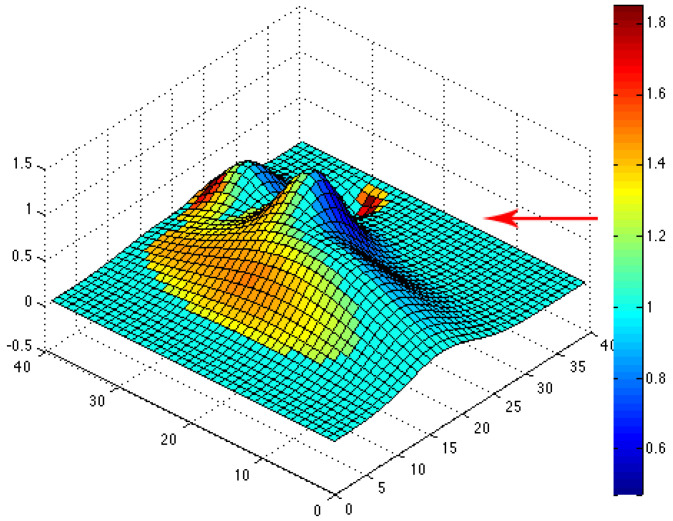
Color map of topographic coefficients associated with a west wind direction. The arrow gives the considered wind direction.

**Figure 8:**
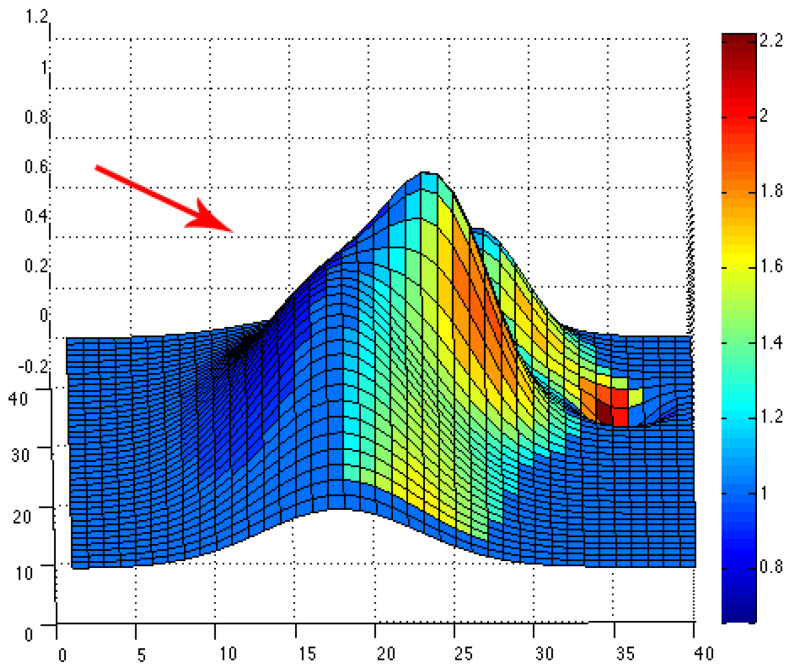
Color map of topographic coefficients associated with a southeast wind direction. The arrow gives the considered wind direction.

The simulated topographical data given by the function f in (10) are then used to compute the topographic coefficients as described in the previous subsection. For each of the eight wind directions, we can plot the color map of the computed topographic coefficients on the obstacle. The associated colors go from dark, for zones where the topography slows down the wind flow (c < 1), to white, for zones where the topography accelerates the wind flow (c > 1). For instance, we have plotted color maps for situations where the wind comes from the northeast (see Figure 6), west (see Figure 7) and southeast (see Figure 8). For each figure, the arrow indicates the direction of the wind.

**Figure 9:**
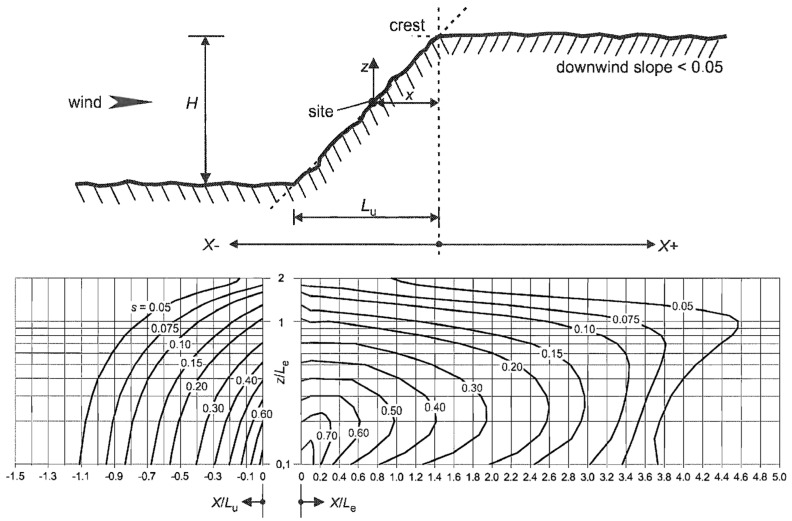
**Top**: case of cliffs or escarpments [13]. **Bottom**: equivalent method to compute orographic coefficients (as we did for hills and ridges).

**Figure 10:**
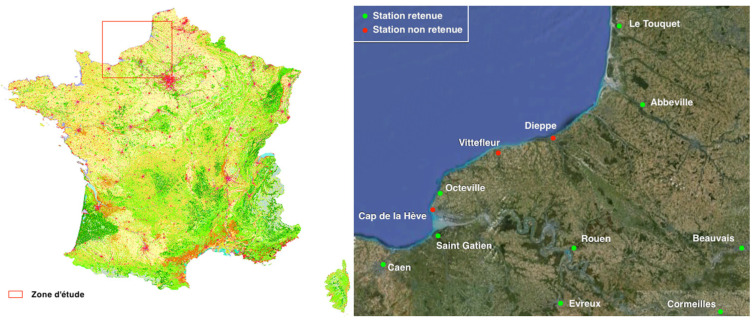
Studied zone (northwest France). Anemometers located in Caen, Octeville, Rouen, Beauvais, Abbeville and Le Touquet were selected.

Note that the function given in (10) permits illustrating the topographic effect in cases where we consider hills and ridges (as in Figure 3). We have tested the topographic effect on more vertical hills and the results were satisfying. For the case of cliffs or escarpments (Figure 9), the computation is slightly different but the reasoning is analogous, and the values of the corresponding orographic parameters are given in Figure 10. An improvement could be to propose calculations to take into account the influence of buildings (vertical walls of buildings with a rectangular plan, the influence of the angle of roofs, etc.) or of vegetation (trees, etc.), especially if we want to reconstruct the wind on a micro-scale.

We now give several numerical experiments on real datasets. The experiments are performed on a 2.21 GHz Athlon with 1.00 GB of RAM.

**Figure 11:**
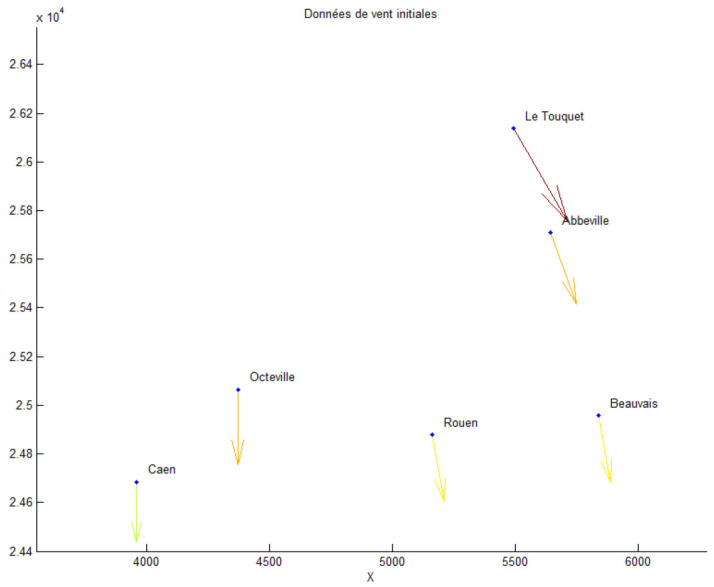
Example of a wind dataset for a given time step. The location is northwest France; the data are from anemometers located at six different airports.

**Figure 12:**
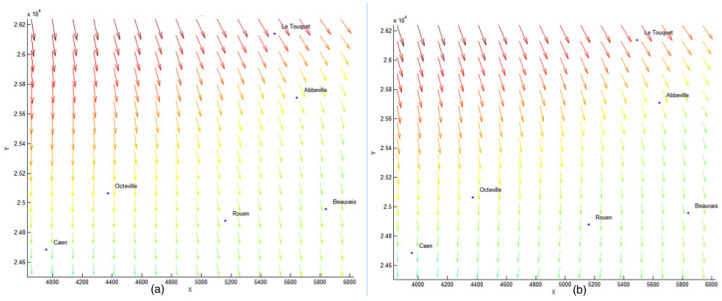
We give two different approximations using the model given in Section 2 of the wind velocity field using a 4 × 4 finite element grid (**a**) and a 3 × 3 finite element grid (**b**). Colors indicate wind speed (same colormap as on Figure 11).

We focus on the data for wind vector fields acquired in northwest France; the dataset takes into account eight weather stations (Meteo France, Figure 10, Figure 11 and Figure 12).

### 4.2. Numerical Simulations of the Global Algorithm

In this subsection, from a set of six velocity wind data, we give the approximation obtained by the method in Section 2, and we then compute the topographic coefficients of the studied zone using the method given in Section 3.

Here is some information about the numerical examples:

- Dataset: six anemometers located at six airports giving the direction and speed of the wind, see Figure 10 and Figure 11;
- Parameter ε = 0.000001;
- Generic finite element: Bogner–Fox–Schmit of class C^1^ (See Appendix A);
- Studied domain: [3500, 6000] × [2.44, 2.62];
- Meshing: 4 × 4 rectangles and 3 × 3 rectangles. The results are given in Figure 12.

The choice of the finite element meshing is crucial, and it must be linked to the number of data we have in the input. For a grid of 3 × 3 rectangles, we have 9 rectangles, 16 nodes and, as we have four basis functions per node with the BFS finite element of class C^1^, the dimension of the space *V_h_* is equal to 64 (while it is 100 with a 4 × 4 meshing, leading to 16 rectangles and 25 nodes). As we do not have a large amount of data, we choose a low number of rectangles in our mesh. If we choose a finer grid, the approximation error increases.

**Figure 13:**
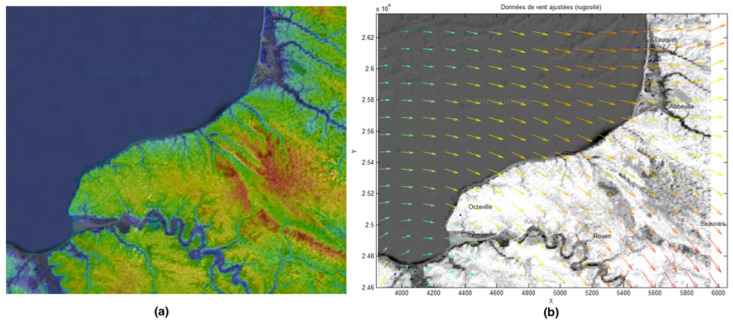
Topographic map of the studied zone (Normandy Region, France) (**a**). Wind vector field (approximated from the six different Meteo France locations at airports) on the topographic map (**b**).

We compute the quadratic error given by the following quotient

$$Quad\_Error={\left(\frac{{\sum_{i=1}^{N}\langle \sigma_{\varepsilon ,h}^{d}(a_{i})-w_{i}\rangle }_{2}^{2}}{\sum_{i=1}^{N}{\langle w_{i}\rangle }_{2}^{2}}\right)}^{1/2},$$

where ${\langle •\rangle }_{2}$ denotes the Euclidean scalar norm. In all our different tests, the quadratic errors is of 10^−3^ and 10^−4^ orders, which is considered as very good in the context of vector field approximation. We then compute this obtained approximation using the topography of the considered zone (see Figure 7 and Figure 13 for the result).

In order to show this method on more complicated datasets, we consider the wind conditions over 90 h; we have the value of the wind vector field at each Meteo France station every 3 h (total of 30 datasets). We apply the previous method for each time step. We then obtain the approximated wind velocity field over the 90 h. We have to propose a way to visualize such datasets.

## 5. Visualization

To obtain a simulation on time using a free library, we first propose using Matplotlib using Python. The following code was developed at INSA Rouen Normandie by the authors (and thanks to H. Merelle from the Applied Math. Department for his help). The main advantage of this code is that it gives a complete framework from the input (dataset) to the numerical simulation, including the approximation using the spline functions, finite element methods and the topography influence.

Algorithm for visualization using Matplolib [14].

Here is the list of files necessary for correct processing and the different steps of the proposed method linked to the flowcharts given in Figure 1:

- Initial Input: dataset (xi, yi, (Ui, Vi))i.
- Definition of Ω—meshing of Ω with rectangles (as we use rectangular finite elements): the number of subdivisions is linked to the number of data; in the examples here, it is 3 × 3 or 4 × 4 subdivisions in x and y.
- D^m^ spline approximation: the output is the evaluation of the vector field on each point of a fine grid of Ω.
- Computation of topography effect on the vector field: output.txt file.
- Script_visualization.py
- The “output.txt” file (in the same folder).

The purpose of the program is to visualize a vector flow from text files, with the possibility of adding a background (topography, etc.).

Data conditions:

- For an animation:

- The “output.txt” file is of the form
  X1 Y1 U1_1 V1_1 U1_2 V1_2…
  X2 Y2 U2_1 V2_1 U2_2 V2_2…
  with X1 and Y1 being the first coordinates, followed by U1_1 V1_1 U1_2 V1_2…; the different sizes of the vectors are a function of time.

- To execute in a terminal under Ubuntu, we use the Python script_visualization.py, with the following instructions:
  ○ The title: it represents the file name (when exporting) and the title of the figure.
  ○ The size of the vector arrows: the bigger they are, the smaller the vectors appear.
  ○ The number of images: if your output file is of the form “Animation”, in this case you will have the following question, “*Enter the number of frames per second*”; it determines the frame rate per second.
  ○ For the background: “O” for accept or “N” otherwise.
  ○ For the name of the image you must give the file extension: here is a non-exhaustive list of usable formats: [name].png, [name].jpg, [name].jpeg and [name].gif.
  ○ To display the result: This command is only used to show you the result. The result is still saved even if you do not display it.
  ○ Data output: For an animation, you can find the animation in the folder in the form [title].gif, and for a fixed image, you can find the rendering in the folder in the form [title].png.

In order to show a numerical simulation, we give a simulation for all of the Normandy region (wind velocity field using the Meteo France dataset) using this Python script and using Matplotlib.

**Figure 14:**
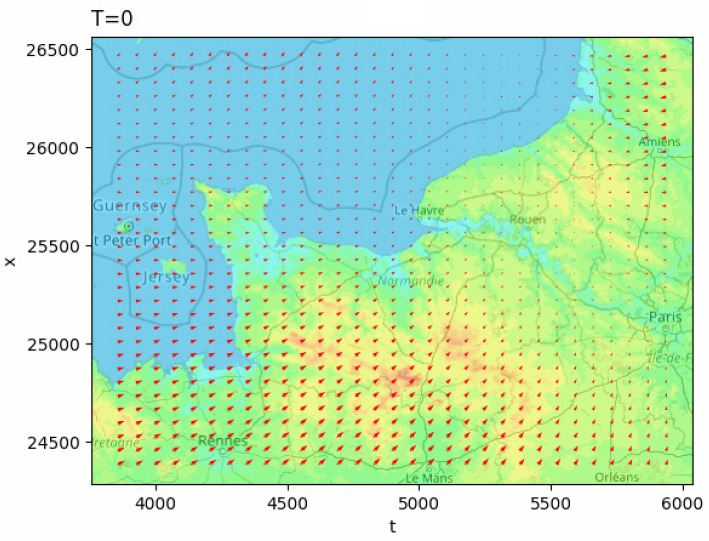
Visualization of a vector flow in Normandy, including the topography effect using Matplotlib (http://lmi.insa-rouen.fr/images/contenu/Movies/Test.gif accessed on 1 November 2024).

**Figure 15:**
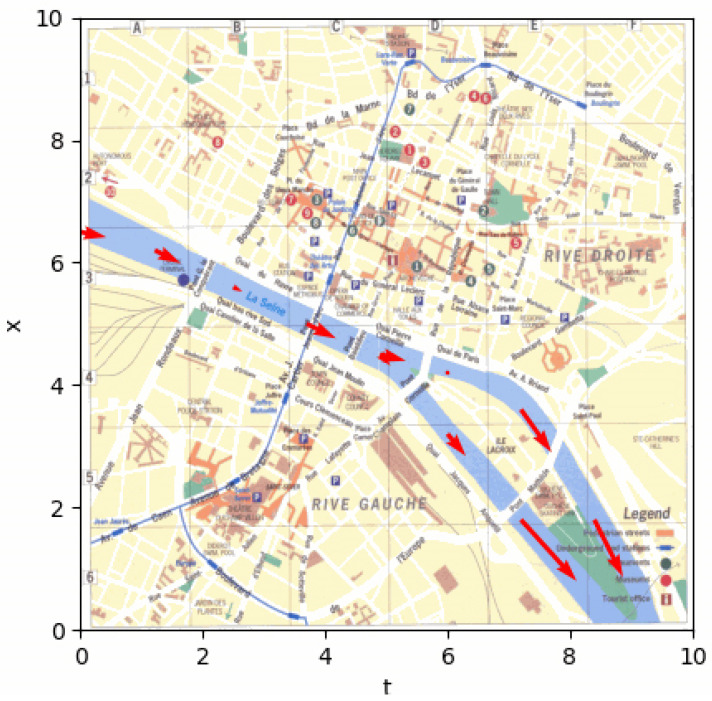
Example of visualization of marine currents in Rouen, France. Arrows indicate directions and speed (following length of the arrow) of the current. Visualization is performed using Matplotlib. (http://lmi.insa-rouen.fr/images/contenu/Movies/Rouen.gif accessed on 1 November 2024).

Examples of the obtained visualizations on a sequence (time) of an approximated velocity vector field (test in the Normandy region, France) and marine current (Seine River at Rouen, France) are given in Figure 14 and Figure 15.

## 6. Discussion and Future Directions

About the approximation method using D^m^ splines, note that a theoretical study of the error in the approximation method following the used finite element mesh is a work in progress. To do that, we use previous results obtained from smoothing spline approximations from a finite set of points, as performed in [10,11]. Another development, linked to the approximation part, will be to propose an automatic meshing of the domain and different choices for the used generic finite element (based on triangles, etc.).

**Figure 16:**
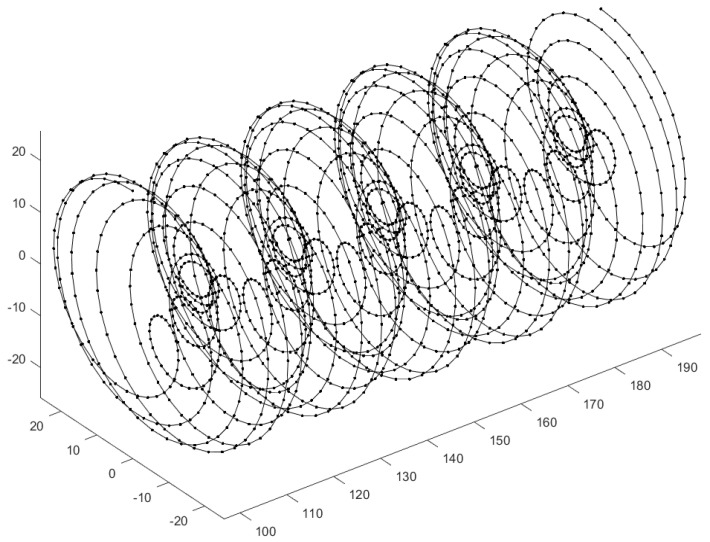
Example of the location of data in a Lidar dataset.

Another goal consists in improving both the modelling and visualization. About the modelling, the goal will be to include new kind of datasets; nowadays, it is possible to obtain wind datasets from Lidar located on wind turbines (see Figure 16). This dataset gives the wind velocity field with a specific geometry: along a spiral. It makes the computation much more difficult because it requires a specific finite elements meshing, which makes the process much less automatic.

**Figure 17:**
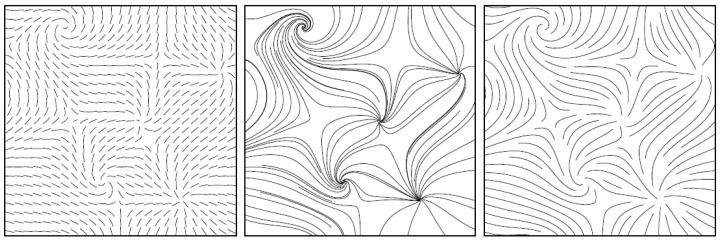
Different streamlines to visualize a vector flow can be used (Short streamlines on the left and long streamlines in the middle. On the right, this is an image we can obtain using the “streamline algorithm” of [15]).

We also plan to add a smooth visualization based on texture using the Matplotlib library. Another crucial point consists in the effect of using a smooth visualization of the flow using streamlines. Vector field data are produced by scientific experiments and numerical simulations, which are now widely used to study complex dynamic phenomena, using a robust method to visualize steady flow field with both line representations and textures. In [15], the authors specify that “a streamline is a line tangential to the vector field at any point. Covering an image with a set of streamlines is a very good way to visualize the flow features” (see Figure 17 and [15] for more details).

In order to show this method on more complicated datasets, we considered the wind conditions over 90 h. We have the value of the wind vector field at each Meteo France station every 3 h (total of 30 datasets equivalent to the one we show in Section 4).

We then computed the obtained vector field using the modelling proposed in this work with the help of B. Jobard [15], and we obtained the movie given in [16]. This result is smooth and promising. But improvements have to be made to propose a tool able to treat the whole process with the same software, and to maybe try other approximation methods (like the one in [17], and to mix this approximation/visualization tool with an image processing framework, or the one in [18,19,20], using radial basis functions; this is ongoing work). Moreover, it is also crucial to develop an algorithm with which to approximate more complex datasets, like Lidar ones (instead of anemometers) to compute wind velocity fields.

## 7. Conclusions

In this work, we successfully proposed a global tool, from vector field approximation to visualization. We proposed a method to obtain a visualization of a vector field from a sparse dataset, after computing its numerical approximation using a mathematical model using energy minimization and finite elements for the discretization. Note that we also integrated the topography effect into the modelling of a wind velocity field approximation method. To our knowledge, this is the first global approach for such numerical simulations from a dataset with few data.

As stressed in Section 6, several developments should occur in the future in order to improve this global approach. Many potential applications exist, from velocity wind approximation for wind turbine energy modelling, current simulation for modelling the morphodynamics of coastal zones and control theory for vehicle navigation (cars, submarines, etc.).

## Figures and Tables

**Table 1:** Features used to compute the characteristic coefficient *s* of an obstacle (source: [13]).

Variables	Definition
*s*	Orographic location factor
*Φ*	Upwind slope *H/L_u_* in the wind direction (see Figure 3)
*L_e_*	Effective length of the upwind side
*L_u_*	Length of the upwind side
*L_d_*	Length of the downwind side
*H*	Effective height of the obstacle
*x*	Horizontal distance between point (x,y) and the top of the obstacle
*z*	Height of the considered point (x,y)

**Figure A1:**
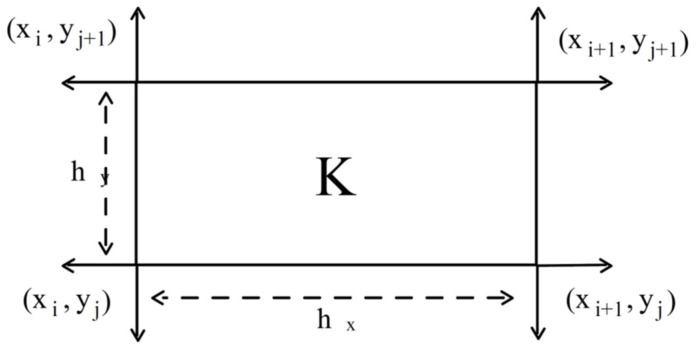
BFS rectangle finite element.

**Figure A2:**
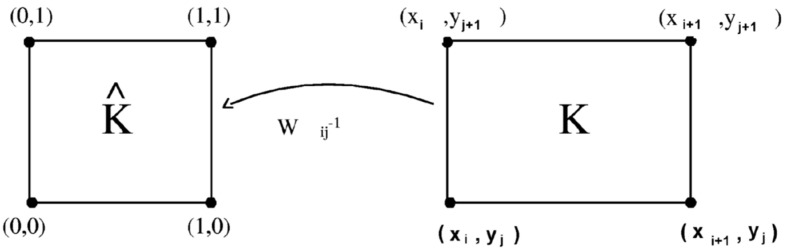
Affine transformation to compute the basis function of any point using the basis function of the reference finite element.

## Data Availability

The original contributions presented in this study are included in the article. Further inquiries can be directed to the corresponding author.

## Appendix A. Bogner–Fox–Schmit Finite (BFS) Element of Class C^1^

We give some results here about the BFS finite element, keeping the usual notations introduced in this field (see [21,22] for more details). We introduce the BFS of class C^1^ we use in this work. We consider a rectangle K, a set of polynomial functions P and a set of degrees of freedom ∑ defined as

- K is the rectangle defined by the four points (*x_i_*,*y_i_*), (*x_i+_*_1_,*y_i_*), (*x_i_*,*y_i+_*_1_) and (*x_i+_*_1_,*y_i+_*_1_) (see Figure A1).
- P = $Q_{3}(R^{2})=\left\{q(x,y)=\sum_{0\leq i,j\leq 3}\alpha_{ij}x^{i}y^{j},\alpha_{ij}\in R\right\}.$
- $\Sigma =\left\{\phi_{kl}:p\mapsto p(x_{k},y_{l});\phi_{kl}^{\left(1\right)}:p\mapsto \frac{\partial p}{\partial x}(x_{k},y_{l});\phi_{kl}^{\left(2\right)}:p\mapsto \frac{\partial p}{\partial y}(x_{k},y_{l}),\phi_{kl}^{\left(3\right)}:p\mapsto \frac{\partial^{2}p}{\partial x\partial y}(x_{k},y_{l})\right\}.$

It easy to check that *dim P = card* ∑ = 16 and that ∑ is P-unisolvant. Thus, the triplet (K, ∑, P) defines a finite element. The 16 elements of ∑ are called the “degrees of freedom” of the considered finite element.

We now have to define the basis function of (K, ∑, P). To do that, we first work on a reference finite element corresponding to the rectangle K = [0, 1] × [0, 1]. Then, an affine transformation gives all the basis functions for all the degrees of freedom of the meshing.

Each basis function is a polynomial belonging to P, with a value of 1 for one of the 16 degrees of freedom, and value of 0 for the 15 others.

For example, on the rectangle of reference [0, 1] × [0, 1], the four basis functions at point (0, 0) are

(A1) $$\begin{array}{l} \phi_{00}(x,y)=(2x+1){\left(x-1\right)}^{2}(2y-1){\left(y-1\right)}^{2}.\\ \phi_{00}^{\left(1\right)}(x,y)=(2x+1){\left(x-1\right)}^{2}y{\left(y-1\right)}^{2}.\\ \phi_{00}^{\left(2\right)}(x,y)=x{\left(x-1\right)}^{2}(2y+1){\left(y-1\right)}^{2}.\\ \phi_{00}^{\left(3\right)}(x,y)=x{\left(x-1\right)}^{2}y{\left(y-1\right)}^{2}. \end{array}$$

We find the 16 basis functions for the 12 other degrees of freedom.

The finite element method then uses the basis function of the “reference” finite element to compute all the basis functions corresponding to the finite element mesh, with four basis functions for each node of the meshing. To do that, we just have to apply a diagonal affine mapping W, such that W([0, 1] × [0, 1]) = K, transforming each vertex of the reference element into one vertex of K (Figure A2). Then, the basis functions of K are trivially obtained using the mapping W and the basis function of the element of reference (see p. 57 of [22] for example).

One of the main advantages of a finite element basis is that these basis functions have a very small support; thus, the matrix of the linear system we obtain is sparse (diagonal and positive definite!). A global introduction to the finite element method can be found in [23].
